# Interactions between Gut Microbiota and Immunomodulatory Cells in Rheumatoid Arthritis

**DOI:** 10.1155/2020/1430605

**Published:** 2020-09-09

**Authors:** Huihui Xu, Hongyan Zhao, Danping Fan, Meijie Liu, Jinfeng Cao, Ya Xia, Dahong Ju, Cheng Xiao, Qingdong Guan

**Affiliations:** ^1^Beijing Key Laboratory of Research of Chinese Medicine on Prevention and Treatment for Major Diseases, Experimental Research Center, China Academy of Chinese Medical Science, Beijing 100700, China; ^2^Institute of Clinical Medicine, China-Japan Friendship Hospital, Beijing 100029, China; ^3^Graduate School of Peking Union Medical College, Chinese Academy of Medical Sciences/Peking Union Medical College, Beijing 100193, China; ^4^School of Traditional Chinese Medicine, Beijing University of Chinese Medicine, Beijing 100029, China; ^5^Department of Emergency, China-Japan Friendship Hospital, Beijing 100029, China; ^6^Cellular Therapy Laboratory, Research Institute in Oncology and Hematology, CancerCare Manitoba, Winnipeg, Canada R3A 1R9; ^7^Department of Immunology & Internal Medicine, University of Manitoba, Winnipeg, Canada R3A 1R9; ^8^Manitoba Centre for Advanced Cell and Tissue Therapy, Winnipeg, Canada R3A 1R9

## Abstract

Rheumatoid arthritis (RA) is one of the most common autoimmune diseases caused by abnormal immune activation and immune tolerance. Immunomodulatory cells (ICs) play a critical role in the maintenance and homeostasis of normal immune function and in the pathogenesis of RA. The human gastrointestinal tract is inhabited by trillions of commensal microbiota on the mucosal surface that play a fundamental role in the induction, maintenance, and function of the host immune system. Gut microbiota dysbiosis can impact both the local and systemic immune systems and further contribute to various diseases, such as RA. The neighbouring intestinal ICs located in distinct intestinal mucosa may be the most likely intermediary by which the gut microbiota can affect the occurrence and development of RA. However, the reciprocal interaction between the components of the gut microbiota and their microbial metabolites with distinct ICs and how this interaction may impact the development of RA are not well studied. Therefore, a better understanding of the gut microbiota, ICs, and their interactions might improve our knowledge of the mechanisms by which the gut microbiota contribute to RA and facilitate the further development of novel therapeutic approaches. In this review, we have summarized the roles of the gut microbiota in the immunopathogenesis of RA, especially the interactions between the gut microbiota and ICs, and further discussed the strategies for treating RA by targeting/regulating the gut microbiota.

## 1. Introduction to RA

Rheumatoid arthritis (RA) is a chronic autoimmune disease that affects approximately 1% of the population worldwide, caused by abnormal immune activation and immune tolerance and characterized by synovial inflammation, cartilage damage, and bone destruction [1]. Previous studies have suggested that immune abnormalities, such as immunomodulatory cell (IC) activation or inhibition, that take place at a local and, subsequently, at a systemic level are present in patients who are at risk of developing RA [2]. The frequency and function of T and B lymphocyte subsets are associated with the pathogenesis of RA [3, 4]. The pathologically increased production of CXCL13 and interleukin- (IL-) 21 by the peripheral T helper (TPH) cell phenotype can recruit T follicular (Tfh) helper cells and B cells, and the TPH cell phenotype can expand the spectrum of B cell-helper T cells within the inflamed synovium of RA patients [5]. T helper 17 (Th17) cells, which can produce IL-17, contribute to the onset of RA [6], while regulatory T (Treg) cells, which secrete the anti-inflammatory cytokine IL-10 and transforming growth factor- (TGF-) *β*1, are pivotal players in the control of RA [7]. Many approaches for the treatment of RA may promote Treg generation/expansion while inhibiting Th17 cell differentiation and consequently restoring the balance of the Th17/Treg cell ratio [8–11]. Some drugs can even decrease the frequency of Th1 cells but increase that of Th2 cells [12]. Autoreactive B cells are also crucial in the aetiology of RA and producing anticitrullinated protein antibodies (ACPAs) and rheumatoid factors (RFs) [3]. In the lymphoid tissue of RA patients, increased T cell activation is related to the persistence of switched memory B cells [13]. A memory B cell subset that expresses the immunoglobulin A (IgA) receptor, termed Fc receptor-like 4 (FcRL4), is a component of the local autoimmune response that can contribute to the joint destruction in RA patients via receptor activation of nuclear factor-*κ*B ligand (RANKL) expression [14]. Numerous immune-related abnormalities in RA notably occur at the mucosal level. During the development of RA, the intestinal mucosal immune response is excessively exaggerated, antigen-presenting cells (APCs) are abnormally activated, and immune tolerance is disturbed. The intestinal mucosal immune system, especially Peyer's patches, is important for inducing immunity tolerance. The higher numbers of CD8^+^ cells and CD4^+^ cells are observed in Peyer's patches of rats with collagen-induced arthritis (CIA) than those of control rats [15]. The CIA animal model shares many clinical, histopathological, and immunological features with clinical human RA, and these similarities are commonly exploited in the use of the CIA model for studying RA [16]. One study showed that in rats with CIA, the concentrations of secretory IgA (sIgA) in the small intestine and interferon-*γ* (IFN-*γ*) in small intestinal tissue homogenates were upregulated, the ratios of CD4^+^/CD8^+^ in both the epithelium and lamina propria of the small intestine were increased, and the expression of CD80, CD86, IL-6, and IL-12 mRNA in the small intestine was also significantly increased compared with those in the control group [17]. Evans-Marin et al. showed that CD4^+^ T cells in the lamina propria were activated before the onset of arthritis in mice with CIA, following the significant upregulation of IL-17A, tumor necrosis factor- (TNF-) *α*, and granulocyte-macrophage colony-stimulating factor (GM-CSF), and the severity of arthritis was significantly reduced if Th17 cells were lacking [18]. Serum analysis of recent-onset RA patients who received antirheumatic therapy showed that the ACPA, sIgA, and IgM levels rapidly decreased, which were related to the decreased disease activity. The decreased mucosal immunity to citrullinated proteins/peptides and the recruitment of new B cells are crucial features of antirheumatic therapy responses in early RA patients [19].

Since genetic factors cannot completely account for the risk of this disease [20], the substantive role of other risk factors needs to be explored. Recently, it has been proposed that environmental factors are potentially involved in RA progression [21]. Additional evidence has shown that the gut microbiota might be an important experimental factor in the occurrence and development of RA [22]. Diverse ICs, especially those located in the intestinal mucosa, are the most likely intermediary by which the gut microbiota can impact the occurrence and development of RA. As a result, there is a possible interaction among the gut microbiota, ICs, and initiation and propagation of RA, which has recently attracted more interest. In this review, we summarize the roles of the gut microbiota in the pathogenesis and treatment of RA, focusing on the interactions between the gut microbiota and intestinal ICs.

## 2. General Introduction to the Gut Microbiota

The gastrointestinal tract of mammals is abundantly colonized by trillions of different prokaryotic microorganisms, including bacteria, archaea, fungi, and viruses, collectively termed the gut microbiota, which have a mutual relationship with their host. The gut microbiota contains 1000-5000 different species of microorganisms that numerically exceed host cells by approximately 10-fold, with 99% belonging to the phyla Firmicutes, Bacteroidetes, Proteobacteria, and Actinobacteria [23–25]. The microbiota composition of the host in adulthood is shaped during the early neonatal period [26]. The microbiota consists of “beneficial” symbiotic microbes that maintain host homeostasis in a cooperative and nonpathogenic manner, “sensitive” microbes that become dysregulated due to diseases, “pathogenic” microbes that can cause disease, and “therapeutic” microbes that can help reverse alterations [27]. The beneficial microbes not only can help to digest and absorb food but also have a protective function that can prevent the adherence of pathogenic microbes to the mucosal layer [28]. Furthermore, the host can provide a niche for the survival of the beneficial microbes. The gut microbiota can be influenced by diet, probiotics, prebiotics, antibiotics, exogenous enzymes, faecal microbiota transplantation (FMT), and other environmental factors [29]. The distribution of the gut microbiota is also affected according to the local regional environment in the gastrointestinal tract [30, 31]. The gut microbiota is necessary for host health, especially for immune homeostasis and function. The components of the gut microbiota play a profound role in modulating the innate and adaptive immunity of the host. The host immune system has the ability to induce immune tolerance towards the existence of beneficial microbes and to prevent the uncontrolled growth of opportunistic pathogens [32]. Commensal microorganisms are required for the development and differentiation of the local and systemic immune systems and nonimmune components [33, 34]. The intestinal barrier plays a critical role in maintaining immune homeostasis via these microorganisms. The gut microbiota participates in immune responses presumably by altering intestinal barrier permeability, modifying self-antigen integrity, mimicking epitopes, and modulating cell apoptosis mechanisms [35–37]. The tolerance to the gut microbiota must be maintained in order for the host to benefit from their coexistence; on the contrary, colonization with specific pathogenic microbes may be detrimental to the host, leading to diseases [33]. The gut microbiota has been found to play important roles in the pathogenesis of many intestinal and extraintestinal diseases [38, 39]. In addition, gut microbiota dysbiosis, which closely interacts with the intestinal mucosal immune system, has been associated with autoimmune diseases such as RA; moreover, the diversity, various taxa from the phylum to genus levels, and associated functions of the gut microbiota are already altered in patients in the early stages of RA [40]. However, the underlying cellular and molecular mechanisms are undefined.

## 3. Interactions of the Gut Microbiota and Metabolites with ICs

The immune system has coevolved with the microbial community inhabiting not only the body surface but also the mucosal barriers [41]. Mucosal sites, such as the oral and intestinal mucosa, may be the sites where autoimmunity is initiated. Although the gut microbiota is spatially confined in the intestinal lumen, these microbes and their related metabolites can shape the function of the host immune system. Some microbial clusters may be harmful and others may be helpful in maintaining the balance of the immune network. The normal immune system is maintained by a balance of protective immunity against pathogens and immune tolerance to self-antigens, and the disruption of this balance may cause the abnormal activation and proliferation of lymphocytes. Recent evidence has highlighted the immunomodulatory effect of the gut microbiota and their metabolites on ICs [42, 43].

### 3.1. Gut Microbiota and ICs

The interaction between the host and gut microbes is partially mediated by intestinal ICs, which sustain a complex balance. Gut microbiota dysbiosis leads to changes in the functionality of intestinal IC subsets [36, 44]. The interplay between the gut microbiota, intestinal epithelium, and innate and adaptive immune cells at homeostasis prevents immune-mediated disease [45, 46]. Gut microbiota may affect intestinal immunity by regulating T cell-mediated mucosal immunity. One of the most prominent functions of the gut microbiota and their metabolites is the regulation of the balance between proinflammatory Th1 and Th17 cells and protective Treg cells at mucosal surfaces and systemically. A recent study demonstrated that *Lactobacillus helveticus SBT2171* (LH2171) directly inhibited the proliferation of lipopolysaccharide- (LPS-) stimulated mouse T and B cells and the cell cycle progression of human lymphoma cell lines (BJAB) in vitro by suppressing the c-Jun N-terminal kinase (JNK) signalling pathway [47]. Segmented filamentous bacteria (SFB) can induce Th17 cell activation [48]. The modulation of cytokine production by APCs may be critical for the anti-inflammatory effects of the gut microbiota. A previous study suggested that *L*. *helveticus SBT2171* (LH2171) could induce the expression of A20 (a negative regulator of NF-*κ*B/MAPK signalling) by Toll-like receptor 2 (TLR2) signalling, thus inhibiting IL-6 and IL-1*β* production by APCs [49]. When autoimmune-prone Dark Agouti (DA) rats were intraperitoneally injected with their own colonic *E*. *coli or Enterococcus* for two days, the proportion of resident and anti-inflammatory macrophages was diminished, the proportion of activated neutrophils was increased, and the inflammatory polarization of peritoneal cells was induced [50]. Tanoue et al. reported that a consortium of 11 bacterial strains isolated from healthy human donor faeces could induce IFN-*γ*-producing CD8^+^ T cells without causing inflammation in the intestine [51].

Various kinds of ICs and their secreted cytokines are necessary to maintain immunological homeostasis and oral tolerance to dietary antigens in the gastrointestinal tract mucosa [52]. The functional actions of ICs require the regulation of gut microbiota, thus establishing a detrimental vicious cycle. The ICs in specific compartments are also regulated in a regional specialization-dependent manner by microorganisms [53]. Colonic epithelial cells consist of different cellular subtypes, including colonocytes, gradients of progenitor cells, and goblet cells within intestinal crypts. The colonic epithelium facilitates interactions between the host and microbiota to form a mucus barrier, control mucosal immunity, and coordinate nutrient recycling through the specific contribution of each epithelial cell subtype [54]. In the small intestine, Zhou et al. showed that group-3 innate lymphoid cells (ILC3s) were the dominant cellular source of IL-2, which was required to maintain Treg; moreover, IL-2 was selectively induced by the IL-1*β* secreted in the small intestine, and activating IL-1*β* production by macrophages required MYD88- and NOD2-dependent sensing of the microbiota [55]. As major innate and adaptive lymphocyte populations, subsets of innate lymphoid cells (ILCs) and conventional T cells sequentially shape the mature commensal gut microbiota and help maintain tissue metabolic homeostasis [56].

### 3.2. Microbial Metabolites and ICs

The gut microbiota produces dozens of metabolites, which can participate in various physiological processes, including the modulation of immune cell function in the host gut mucosa [57]. As one of the active microbial metabolites, short-chain fatty acids (SCFAs) can induce metabolic alterations in T cells by activating the mTOR complex and modulating glucose metabolism [43]. Butyrate, a functional SCFA produced by the anaerobic gut microbiota, has been shown to prevent CIA in mice; butyrate might mediate the differentiation of CD4^+^ T cells towards Treg cells in the spleen, increase systemic Treg cells and decrease systemic Th17 cells, and enhance the polarization of Treg cells but not that of Th17 cells [58]. Shen et al. showed that antibiotic treatment could reduce the abundance of the microbiota in the colon, which led to decreased proportions of Treg cells and SCFAs in IL-10-deficient mice with colitis [59]. The bacterial metabolites pyruvic acid and lactic acid, which are produced in a bacteria-dependent manner, can lead to enhanced immune responses by GPR31-mediated induction of dendrite protrusions in small intestinal mononuclear cells that express CX3CR1 (CX3CR1^+^ cells) [60]. The aryl hydrocarbon receptor (AhR), which is a ligand-dependent transcription factor, can recognize not only tryptophan metabolites but also endogenous microbiota-derived factors and dietary components [61]. AhR can mediate crosstalk between ILCs and other immune cells in host tissues, especially in the intestinal mucosal surface [62], which may be the bridge between the gut microbiota and ICs. Li et al. demonstrated that human umbilical mesenchymal stem cells played a therapeutic role in rats with CIA by modulating the interactions between the immune status of Treg, Th17 cells, and B cells and the gut microbiota in the ileum via AhR [63].

## 4. Gut Microbiota and Metabolites in RA

Because of the interactions between the gut microbiota and host homeostasis, the former is believed to trigger RA through the regulation of ICs, which are both near to and distant from the site where they occur to induce. Recent advances have shown that the complex interaction between the genetic and environmental factors can contribute to the aetiology of RA [64]. However, the mechanisms underlying the outcomes still need to be elucidated. Lu et al. reported that mice deficient in *TYRO3/AXL/MER* (TAM) receptors spontaneously developed clinical characteristics of RA at the age of 6 months [65], but a subsequent study showed that TAM triple knockout (TKO) mice did not spontaneously develop any macroscopic arthritis-like symptoms until 52 weeks of age [66]; this difference might be due to an interplay between the genetic and environmental factors. A previous study found that the human leukocyte antigen (HLA) alleles HLA-B27 and HLA-DRB1 could favour a more inflammatory gut microbiome and/or aberrant immunologic responses to bacteria, which drive the immunopathogenesis of RA [67]. The gut microbiota and microbial metabolites strongly interact with many RA-related genes and affect RA-associated immune pathways and immunological phenotypes [68]. Observations in patient studies and in animal model experiments about RA have revealed that alterations in the host gut microbiota can influence susceptibility to onset and subsequent progression of RA (Table 1).

### 4.1. Gut Microbiota Dysbiosis Triggers Inflammatory Arthritis in Human RA Patients

A certain number of bacterial species and metabolite profiles may characterize some type of RA and predict RA progression. Chen et al. showed that the abundance of *Collinsella* segregated with RA was correlated with high levels of *α*-aminoadipic acid and asparagine and with the secretion of IL-17A. The *Collinsella* increased gut permeability by reducing the expression of tight junction proteins in the human epithelial cell line CACO-2 in vitro, which suggested that the expansion of *Collinsella* enhances proinflammatory conditions through a loss of gut epithelial integrity [69]. A previous study showed an enrichment of the bacterial family Prevotellaceae, particularly *Prevotella* spp., in the “preclinical RA” group compared with that in the first-degree relative (FDR) (asymptomatic patients without autoantibodies) control group [70]. The abundance of *Prevotella* in some early RA patients is higher compared with that in the control group [71]. Pianta et al. revealed that subgroups of RA patients had differential IgG or IgA immune reactivity against *P*. *copri*, which was associated with Th17 cytokine responses and frequent ACPAs, so they suggested that *P*. *copri* is immunologically relevant to RA pathogenesis [72]. The presence of multiple *Prevotella* spp. in the gut microbiota, in addition to *P*. *copri*, was associated with RA aetiology by metagenome-wide shotgun sequencing [73]. Studies showed that compared to that of healthy controls, the faecal microbiota of RA patients contained more *Lactobacillus* communities, according to the higher richness, Shannon-Wiener, and evenness measures results [74], and had more members of the phylum Verrucomicrobiae and the genus *Akkermansia*. Interestingly, a higher abundance of Enterobacteriaceae and *Klebsiella* and a lower abundance of *Bifidobacterium* were detected in RA patients who had high serum levels of TNF-*α* or IL-17A [75]. In RA patients, the phylum Euryarchaeota was directly associated with the disease activity score on 28 joints (DAS-28) and emerged as an independent risk factor, and patients treated with etanercept (ETN) presented a partial restoration of the gut microbiota (Cyanobacteria, the class Nostocophycideae, and the order Nostocales increased, while the class Deltaproteobacteria and the family Clostridiaceae decreased) [76]. *Haemophilus* spp. were decreased in RA patients and negatively correlated with the levels of serum autoantibodies [77]. A recent study found that there was an increase in *Bacteroides* and *Escherichia-Shigella* and a decrease in *Lactobacillus*, *Alloprevotella*, *Enterobacter*, and *Odoribacter* in RA patients. Furthermore, using the Spearman correlation analysis, *Dorea* and *Ruminococcus* were positively correlated with RF-IgA and anti-CCP antibodies, and *Alloprevotella* was positively correlated with numerous rheumatoid factors, such as RF-IgM, RF-IgA, and RF-IgG, and with inflammatory biomarkers, including the erythrocyte sedimentation rate and C-reactive protein [78]. By assessing the freshly collected faecal samples from RA patients, Ebrahimi et al. reported that the serum levels of RF, ESR, CRP, anticyclic citrullinated peptide (anti-CCP), and antimutated citrullinated vimentin (anti-MCV) were significantly upregulated in *Helicobacter pylori*- (H. pylori-) positive patients compared with those in *H*. *pylori*-negative patients [79].

The comparison of patients with RA and comorbidities mediated by the gut microbiota has also been studied. A potential microbial link for inflammatory arthritis may exist in patients with RA and inflammatory bowel disease- (IBD-) associated arthropathy because these patients share a higher abundance of Clostridiaceae than nonarthritic controls [80]. The gut microbiota is also different between RA and osteoarthritis (OA) patients. Lee et al. demonstrated that RA patients had a lower relative abundance of *Bacteroides* and *Bifidobacterium* and a lower Bacteroidetes : Firmicutes ratio than OA patients and that the abundance of certain bacterial species, including *Fusicatenibacter saccharivorans*, *Dialister invisus*, *Clostridium leptum*, *Ruthenibacterium lactatiformans*, *Anaerotruncus colihominis*, *Bacteroides faecichinchillae*, *Harryflintia acetispora*, *Bacteroides acidifaciens*, and *Christensenella minuta* [81], was significantly lower in RA patients.

### 4.2. Gut Microbiota Dysbiosis Triggers Inflammatory Arthritis in CIA

The CIA animal model is a common experimental animal model of RA that is established by immunization of animals with type II collagen, and this model has clinical features similar to those of human RA. The interplay between the gut microbiota and inflammatory arthritis in animal models has been demonstrated in vivo [82]. Previous studies reported that the alteration of the gut microbiota composition marked the preclinical phase of murine CIA and preceded the development of disease [83, 84]. Compared with nontreated mice, CIA-susceptible HLA-DQ8 mice treated with *C*. *aerofaciens* increased the incidence and severity of arthritis [69]. Liu et al. showed that the microbial richness and diversity were different between mice that were susceptible and resistant to CIA before the initiation of arthritis. With the progression of CIA, the abundance of the operational taxonomic units (OTUs) affiliated with the families Bacteroidaceae, Lachnospiraceae, and S24-7 was markedly upregulated in CIA-susceptible mice; if germ-free mice were administered the microbiota from either CIA-susceptible or CIA-resistant mice, the former group presented a higher incidence of arthritis with increased IL-17 levels and CD8^+^ T cell and Th17 lymphocyte proportions but decreased dendritic cells (DCs), B cells, and Treg cells in the spleen than the latter group [85]. The immune response and the gut microbiota profiles occur at different stages of CIA. Nemoto et al. reported that the percentage of Foxp3^+^CD4^+^ T cells was only increased in the mesenteric lymph nodes (MLNs) in the relapse stage of CIA. The percentage of ROR*γ*^+^CD4^+^ T cells was increased in the MLNs at the initial peak and was decreased in the relapse stage of CIA, but the opposite changes were observed in the spleen. The concentration of IgA in the faeces increased with the progression of arthritis and showed positive correlations with Bacteroidales in the CIA group [86]. Gut commensal bacteria can regulate gut immunity. Balakrishnan et al. showed that RA-associated bacteria (*Eggerthella lenta* or *Collinsella aerofaciens*) enhanced gut permeability in DQ8 mice. The splenocytes from naive DQ8 mice gavaged with *E*. *lenta* produced the proinflammatory cytokines IL-6, IL-21, and IL-23. When compared with the controls, DQ8 mice gavaged with non-RA-associated bacteria (*Prevotella histicola* or *Bifidobacterium* sp.) exhibited decreased numbers of inflammatory monocytes and CD11c^+^Ly6c^+^ cells and reduced levels of proinflammatory monocyte chemotactic protein- (MCP-) 1 and MCP-3 [87]. In a recent metabolomic study, altered metabolites contained citric acid and l-isoleucine both in the serum and in the faeces were identified in rats with CIA compared with the control rats [88]. Jubair et al. found that in the CIA model, the absence of a dominant microbiota resulted in an approximately 40% reduction in disease severity through the regulation of mucosal and systemic cytokines and autoantibodies, and gut microbial dysbiosis was associated with the mucosal Th17 immune response, stimulating mucosal lymphoid tissue-producing autoantibodies and regulating autoantibody effector functions in the preclinical phase of CIA [89]. Maeda et al. suggested that T cells from germ-free, arthritis-prone SKG mice were activated in the intestine by dysregulated microbiota from RA patients, which caused joint inflammation [90]. Certain antibiotics can induce sustained changes in gut immunity by increasing the ratios of Th1 and Th17 cells in the MLNs, which might be responsible for the aggravation of CIA and attributed to the disruption dysregulation of microbes [91].

Whether the dysregulation of the gut microbiota precedes the onset of RA or is a consequence of RA still needs to be investigated. Previous research showed that the gut microbiota could be changed with the aggravation of CIA by orally administering *Porphyromonas gingivalis* [92]. Hablot et al. suggested that concomitant experimental colitis in mice with CIA could slightly delay arthritis onset and reduce arthritis severity, which was associated with changes in the gut microbial composition [93]. The difference in the faecal microbial composition was correlated with disease severity in CIA mice [94]. The partial depletion of the natural gut microbiota, which was observed in the colonic content, could aggravate CIA symptoms and increase in vitro Th1/Th17 cytokine production by axillary lymph node cells (ALNCs) from arthritic mice treated with antibiotics compared to the positive control [95]. However, in contrast to these findings, Wing et al. showed that the increased CIA susceptibility of germ-free, reactive oxygen species- (ROS-) deficient Ncf1 mutant mice was not dependent on commensal bacteria when they were reared in specific pathogen-free (SPF) conditions [96]. These differences may be attributed to the distinct local bacteria in the respective SPF facilities where the different experiments were conducted.

### 4.3. Gut Microbiota in Distinct Compartments of the Gastrointestinal Tract Plays Region-Specific Roles in RA (and CIA)

Gut microbiota dysbiosis could contribute to the onset and development of RA, and one of the mechanisms may be through the disruption of the proportions and activation status of local ICs along the intestine, including the dysregulation of effector and regulatory ICs. Microbial analyses of stool samples have been accepted as methods to explore the relationship of the gut microbiota with RA and CIA [98]. However, functional heterogeneity of distinct gastrointestinal tract segments gives rise to regional differences in the gut microbiota [30], with its components that closely interact with their neighbouring ICs, and both display regional specificity within the same individual [99]. The lower gastrointestinal tract comprises a variety of different microbiota along the small intestine, caecum, and colon. The small intestine contains lower microbial diversity than the caecum and colon [100]. Asquith et al. showed that the microbiota composition was strikingly different in stool samples compared to that in mucosal samples, and there was also a marked difference in the ileal site compared with the colonic site [67]. In addition, a less diverse bacterial population was found in the ileum (mucosa and lumen) than that in the caecum of broiler chickens, by terminal restriction fragment length polymorphism (T-RFLP) analysis and sequence analyses of 16S rRNA genes [101]. Another study reported that early antibiotic exposure in suckling piglets changed the abundance of the gut microbiota and its related metabolites in the ileal digesta, which were different from those in the caecal digesta [102]. In aged rats, Lee et al. found that the *β*-diversity of the microbiota was higher in the ileum than in the caecum, but the *α*-diversity of microbiota composition was higher in the caecum than in the ileum. The family Lactobacillaceae was more enriched in the ileum than in the caecum, while Ruminococcaceae and Lachnospiraceae were more abundant in the caecum [31]. Doonan et al. demonstrated that the gut microbiota showed different dysregulations between ileal and colonic contents of mice with CIA; the former exhibited outgrowths of Firmicutes and Proteobacteria in the ileum and decreased Firmicutes with a compensatory increase in Bacteroidetes in the colon, and treatment with ES-62 increased the overall species diversity in the ileum but not in the colon of mice with CIA [103]. Thus, it may not be sufficient to measure faecal samples in order to explore the mechanisms of RA that are mediated by the numerous intestinal microbes in the host.

The large intestine and the small intestine have different functions, in which the functions of the large intestine are mainly the extraction of water and salt from solid wastes, while the main functions of the small intestine are the absorption of nutrients and minerals contained in the diet. In addition, the distinct regions of the intestinal tract not only exhibit different functions but also possess different properties of immune cells with distinct roles in immune modulation [53]. The human colon and ileum contain DCs with distinct roles in mucosal immunity; thus, they should be regarded as separate entities. Mann et al. indicated that a lower proportion of colonic DCs produced TNF-*α* and IL-1*β* when compared with their (paired) ileal counterparts and that colonic DCs exhibited an enhanced ability to induce the expansion/development of CD4^+^FoxP3^+^IL-10^+^ T cells (Treg). It also demonstrated that colonic and ileal DCs have different abilities to imprint homing properties on T cells [104]. ROR*γ*^+^ Treg, as a unique microbe-responsive cell type, were detected in lower proportions in the small intestine compared with those in the colon [105]. A recent analysis of T cells from i*Foxp3* mice after 5 weeks of tamoxifen pulse labelling demonstrated a significant reduction in the frequency and number of CD4^+^Foxp3^+^ T cells in most of the analysed compartments (including MLNs, small intestine epithelium, and lamina propria), except the large intestine, but found an accumulation of intraepithelial CD4^+^ in the small intestine. The plasticity of the Treg cells in the epithelium is microbiota dependent, and these cells exhibit intratissue specialization, which is shaped by discrete niches in the intestine [106]. The follicle-associated epithelium (FAE) of the human ileum, which includes Peyer's patches, is functionally distinct from the regular villus epithelium (VE) because the former is more prone to bacterial-epithelial cell interaction and antigen delivery to the mucosal immune system [107]. The partial elimination of the gut microbiota during established CIA modulates the mucosal T helper cell balance, which mainly presents differently in distinct tracts of the intestine. Broad-spectrum antibiotic (ABX) treatment of mice with CIA significantly reduced the expression of IL-17 mRNA in the terminal ileum, which is a main site for the microbiota-induced T cell modulation, while ABX treatment did not affect the expression of IL-17 in the colon. In addition, the expression of the Treg-related transcription factor FoxP3 was significantly upregulated in the colon tissue of ABX-treated mice, but it was not affected in the small intestine [83]. A proteome analysis study found that the colon mucosa could trigger the production of ACPAs, which is known to contribute to the onset of RA [108]. Therefore, the interplay of the gut microbiota and intestinal ICs may participate in RA in a special tissue-dependent manner that is shaped by discrete compartments in the intestine. Observations of the compartmentalization of the ICs and gut microbial species along specific regions of the intestine at a steady state in RA may provide a platform for understanding the pathogenesis of this disease. Therefore, characterizing the microbial alterations in distinct intestinal tracts during established arthritis may be suggested.

## 5. Targeting the Gut Microbiota for the Treatment of RA

Recent studies have proposed that the commensal microbiota is one of the important environmental triggers of RA due to its interactions with the host immune system [2]. Therefore, immunosuppressive agents that can restore the gut microbial composition and immunologic balance may act as therapeutic drugs for inflammatory arthritis (Table 2).

### 5.1. Gut Microbiota Mediate the Effect of Drugs on RA

The gut microbial composition is quite different between RA patients who received disease-modifying antirheumatic drugs (DMARDs) and healthy controls. Rodrigues et al. found that relative expression units (REU) of *Bacteroides* and *Prevotella* species were increased and REU of *Clostridium leptum* in the faecal samples from RA patients receiving DMARDs were decreased compared with those in samples from healthy controls [97]. The administration of clindamycin for 4 weeks increased the incidence and severity of CIA in mice, and the abundance of anaerobic bacteria was significantly decreased compared to that observed after vancomycin administration [91]. Yue et al. demonstrated that oral administration of berberine can ameliorate symptoms in rats with CIA by downregulating the diversity and richness of gut bacteria, including the abundance of *Prevotella*, and upregulating the abundance of butyrate-producing bacteria to increase the generation of butyrate and stabilize intestinal hypoxia and nitrate supply [109]. Doonan et al. indicated that the subcutaneous administration of ES-62 (an immunomodulator secreted by tissue-dwelling *Acanthocheilonema*) could protect against joint disease in mice with CIA, which was associated with the normalization of the gut microbiota and the prevention of intestinal barrier integrity loss [103]. *Paederia scandens* extract (PSE) effectively inhibited paw swelling, tissue fibrosis, and inflammatory cell infiltration and decreased the serum levels of TNF-*α*, IL-1*β*, IL-6, IL-7, and IL-23 in mice with CIA. Moreover, PSE treatment restored the gut microbial ecosystem of mice with CIA by decreasing the relative abundance of inflammation-related microorganisms, including *Desulfovibrio*, *Mucispirillum*, *Helicobacter*, and Lachnospiraceae [110]. A long-term experiment indicated that the gut microbiota might play a profound role in mediating the therapeutic effects of total glucosides of paeony (TGP) in rats with CIA because of its capability to significantly reverse the taxonomic changes in this animal model, to increase the relative abundance of beneficial symbiotic bacteria, and to inhibit the levels of intestinal cytokines, sIgA and IFN-*γ* [111]. The oral administration of the gut microbial metabolites SCFAs ameliorated the severity of CIA, which was associated with inhibiting Th1 cells but promoting Treg cells [112]. Guo et al. reported that total clematis triterpenoid saponins (CTSs) in CIA rats could improve arthritis symptoms and significantly downregulate the total SCFA concentration, and by LEfSe and DESeq2 analyses, the CTSs could restore the most significantly increased Gram-negative (G(-)) and decreased Gram-positive (G(+)) genera [113].

Certain formulas of traditional Chinese medicine (TCM) also have an effect on RA by impacting the gut microbiota. Qingluo Tongbi decoction (QLT) had a beneficial effect on altered bacterial genera and families (Lachnospiraceae, Eubacteriaceae, and Leuconostocaceae) in rats with adjuvant-induced arthritis (AA) and significantly decreased the expression levels of cadherin-11, IL-17*α*, TLR2, and TLR4 in synovial tissues, which negatively correlated with the abundance of *Staphylococcus* and *Candidatus_Saccharimonas* [114]. The families of bacteria in the faeces and the metabolites in both the serum and faeces were altered in rats with CIA, and the Zushima tablet (ZT) restored most of these metabolites (including l-isoleucine, l-aspartic acid, pyruvic acid, cholic acid, and hypoxanthine) and bacteria (such as Coriobacteriaceae, Bacteroidaceae, and Porphyromonadaceae) [88]. The route of administration may also influence the effect of drugs mediated by the gut microbiota and their metabolites on animals with CIA. It has been reported that kaempferol treatment administered intragastrically rather than intraperitoneally could restore the gut microbiota composition and regulate the microbial metabolism (energy production and tryptophan, fatty acid, and secondary bile acid) in CIA mice and might be responsible for the antiarthritis effects [115].

### 5.2. Probiotics Would Be a Potential Therapy for RA

The gut microbiota might serve as a therapeutic target for many kinds of diseases. Restoring the aberrant gut microbiota to the healthy state is a potential therapeutic approach for preventing RA. At present, resetting gut microbial dysbiosis through probiotics, prebiotics, or FMT is emerging as a potential approach for the prevention and treatment of RA. Probiotics are nonpathogenic microorganisms that can interact with the gut microbiota and provide benefits for the host. The treatment of rats with AA with *Lactobacillus casei* (*L. casei*, ATCC334) could inhibit joint swelling, decrease arthritis scores, improve bone destruction, restore some *Lactobacillus* strains to normal, and decrease the expression of the proinflammatory cytokines IFN-*γ*, TNF-*α*, IL-1*β*, IL-17, and IL-6 [116]. The oral administration of *L*. *casei* had an antiarthritic effect and inhibited cyclooxygenase- (COX-) 2 by decreasing proinflammatory cytokines in a model of CIA [117]. Liu et al. demonstrated that the administration of *L*. *salivarius* UCC118 and *L*. *plantarum* WCFS1 isolated from RA patients could alleviate arthritis in mice with CIA, decrease Th17 cells, and increase Treg, and *L*. *salivarius*-treated mice with CIA also showed a significant increase in the anti-inflammatory IL-10 serum levels [118]. Esvaran et al. reported that the administration of *L*. *fermentum PC1* could markedly reduce paw inflammation and synovial infiltration, attenuate cartilage damage, decrease the proinflammatory cytokine IL-12, and increase the anti-inflammatory cytokines IL-4 and IL-10 in DBA/1 mice with CIA [119]. Yamashita et al. suggested that both the oral administration and intraperitoneal injection of *L*. *helveticus SBT2171* could prevent the CIA symptoms of mice and decrease the subsequent production of bovine type II collagen- (bCII-) specific antibodies; in addition, intraperitoneal injection of *L*. *helveticus SBT2171* could also reduce the numbers of immune cells, including the total B cells, germinal centre B cells, and CD4^+^ T cells, in the draining lymph nodes and the serum level of IL-6 [120]. Oral administration of *L*. *delbrueckii* subsp. *bulgaricus OLL* 1073R-1 prevented CIA in DBA/1J mice, inhibited the secretion of proinflammatory cytokine IFN-*γ* by lymph node cells in response to bCII, and reduced the IL-6, TNF-*α*, and MCP-1 produced by accessory cells [121, 122]. Moreover, probiotic administration in RA may reduce organ damage to a greater extent than nonsteroidal anti-inflammatory drugs (NSAIDs). In Wistar rats with CIA, *L*. *acidophilus* decreased arthritis scores and maintained the normal histology of reproductive organs and the oxidative stress parameters in ovaries and testes [123]. Hosoya et al. reported that the intraperitoneal administration of *L*. *helveticus* SBT2171 (LH2171) alleviated CIA symptoms in DBA/1J mice, and the mechanisms might be the inhibition of excessive lymphocyte proliferation and the generation of immunosuppressive effects in vivo [47]. The oral administration of *L*. *casei* suppressed arthritis severity in rats with CIA, which was associated with the more effective inhibition of CII-reactive Th1-type IgG isotypes (IgG2a and IgG2b), the more effective promotion of IL-10 levels, and the reduction in proinflammatory molecules (such as IL-1*β*, IL-2, IL-6, IL-12, IL-17, IFN-*γ*, TNF-*α*, and COX-2) by CD4^+^ T cells [124]. The systemic administration of exopolysaccharide (EPS), derived from *L*. *rhamnosus KL37*, could ameliorate active CIA induced by the systemic injection of collagen and lipopolysaccharide via the inhibition of arthritogenic CII-specific antibody production [125]. In addition, dietary interventions targeting the microbiota may become a potential therapy for RA. A recent study proved that after 28 days of intervention with a high-fibre diet, RA patients had increased circulating Treg cell numbers, favourable Th1/Th17 ratios, and improved symptoms, which might be due to the regulation of the gut microbiota and microbial metabolites [126].

## 6. Perspective and Conclusion

**Figure 1:**
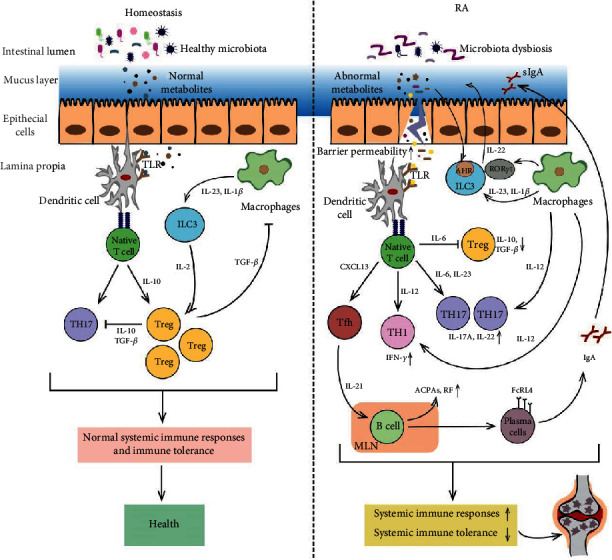
Gut microbiota dysbiosis contributes to the pathogenesis of RA, and a variety of ICs act as a bridge linking the gut microbiota and RA via multiple molecular mechanisms. The healthy gut microbiota and their normal metabolites maintain the integrity of the intestinal epithelial cell layer and the homeostasis of gut immunity.

The interactions between the gut microbiota and ICs, especially intestinal ICs, may hold the keys for developing novel biomarkers and treatment strategies as well as for understanding the pathophysiology of RA (Figure 1). However, the mechanisms of the alteration of specific gut microbiota clusters in RA need to be further investigated. Studies are also required to explore whether some specific gut microbial dysbiosis is either an active driver of RA or is only an epiphenomenon. Furthermore, the potential molecular mechanisms of the therapeutic actions of probiotics for RA remain unclear. Additional studies need to be conducted to explore the involvement of the gut microbiota in distinct intestinal tracts in immune modulation during RA pathogenesis.

## Figures and Tables

**Table 1:** The alteration of the gut microbiota in RA patients and animal models.

Study objects	Sample type	Technology	Increased	Decreased	References
Human, compared RA patients with nonarthritic controls	Stool	Metagenomic shotgun sequencing	Clostridiaceae		[80]
Human, compared RA patients with osteoarthritis patients	Stool	16S ribosome (r)RNA sequencing	*Lactobacilli*, *Prevotella*	*Bacteroides*, *Bifidobacterium*, Bacteroidetes/Firmicutes	[81]
Human, compared RA patients with healthy controls	Stool	16S rRNA sequencing	*Bacteroides*, *Escherichia*-*Shigella*	*Lactobacillus*, *Alloprevotella*, *Enterobacter*, *Odoribacter*	[78]
Human, compared RA patients with healthy controls	Stool	Whole-genome shotgun sequencing	*Prevotella*		[73]
Human, compared RA patients with healthy controls	Stool	16S rRNA sequencing	Verrucomicrobiae, *Akkermansia*		[75]
Human, compared preclinical RA patients with first-degree relatives (FDR) of RA patients	Stool	16S rRNA sequencing	Prevotellaceae, *Prevotella* spp.		[70]
Human, compared FDR of RA patients with healthy controls	Stool	16S rRNA sequencing	*Collinsella*	Actinobacteria	[69]
Human, compared RA patients with healthy controls	Stool	Metagenomic shotgun sequencing	*Lactobacillus salivarius*	*Haemophilus* spp.	[77]
Human, compared RA patients with healthy controls	Stool	qPCR	*Bacteroides*, *Prevotella*	*Clostridium leptum*	[97]
Mouse, compared mice with CIA at the initial peak and relapse of arthritis with healthy controls	Stool	16S rRNA gene sequencing	*Bacteroides*, Bacteroidales	Firmicutes	[86]
Mouse, compared mice with CIA with healthy controls	Stool	16S rRNA gene sequencing	Clostridiales, Deferribacterales, *Mucispirillum*	Enterobacteriales	[94]

RA: rheumatoid arthritis; CIA: collagen-induced arthritis.

**Table 2:** The partial restoration of the gut microbiota in the treatment of arthritis.

Objects and therapy	Samples	Technology	Increased	Decreased	References
Human, etanercept group vs. naive patient group	Stool	Metagenomic sequencing	Cyanobacteria, Nostocophycideae, Nostocales	Deltaproteobacteria, Clostridiaceae	[76]
Mice, kaempferol group vs. CIA group	Stool	16S rRNA sequencing	Bacteroidales_S24-7_group, Prevotellaceae, Erysipelotrichaceae, Alcaligenaceae	Lachnospiraceae, Staphylococcaceae	[115]
Mice, ES-62 group vs. CIA group	Ileal and colonic content	Metagenomic shotgun sequencing	Clostridaceae, Lachnospiraceae (in the ileum)	*Helicobacter*, *Escherichia* (in the colon)	[103]
Mice, PSE group vs. CIA group	Stool	16S rRNA sequencing	Bacteriodetes, *S24-7*, *Rikenella*	*Desulfovibrio*, *Rikenellaceae_RC9*, *Mucispirillum*, *Helicobacter*, Lachnospiraceae	[110]
Rats, berberine group vs. CIA group	Caecal content	16S rRNA sequencing	*Blautia*, *Butyricicoccus*, *Parabacteroides*	*Prevotella*, *Paraprevotella*, *Coprococcus*	[109]
Rats, TGP group vs. CIA group	Stool	16S rRNA sequencing	*Tenericutes*, *Mollicutes*, *Mollicutes RF9*, *Christensenellaceae*, *Unclassified_Erysipelotrichaceae*, *Anaerovorax*		[111]
Rats, QLT group vs. AA group	Stool	16S rRNA gene sequencing	*Ruminococcus_1*, *Clostridium_sensu_stricto_1*, *Atopostipes*, *Turicibacter*, *Ruminococcaceae_UCG-013*, *Roseburia*	*Anaerofustis*, *Blautia*, *Parasutterella*, *Leuconostoc*	[114]
CIA rats, ZT group vs. CIA group	Stool	16S rRNA gene sequencing	Coriobacteriaceae	Bacteroidaceae, Porphyromonadaceae	[88]
AA rats, *L*. *casei-treated* group vs. AA group	Stool	Metagenomic sequencing	*Acinetobacter* unclassified, *Corynebacterium casei*, *L*. *acidophilus*	*Corynebacterium urealyticum*, *Desulfovibrio desulfuricans*, Erysipelotrichaceae	[116]

CIA: collagen-induced arthritis; AA: adjuvant-induced arthritis; PSE: *Paederia scandens* extract; TGP: total glucosides of paeony; QLT: Qingluo Tongbi decoction; ZT: Zushima tablet.
